# Breaking up the Wall: Metal-Enrichment in Ovipositors, but Not in Mandibles, Co-Varies with Substrate Hardness in Gall-Wasps and Their Associates

**DOI:** 10.1371/journal.pone.0070529

**Published:** 2013-07-24

**Authors:** Carlo Polidori, Alberto Jorge García, José L. Nieves-Aldrey

**Affiliations:** 1 Departamento de Biodiversidad y Biología Evolutiva, Museo Nacional de Ciencias Naturales-Consejo Superior de Investigaciones Científicas, Madrid, Spain; 2 Laboratorio de Microscopia, Museo Nacional de Ciencias Naturales- Consejo Superior de Investigaciones Científicas, Madrid, Spain; Consiglio Nazionale delle Ricerche (CNR), Italy

## Abstract

The cuticle of certain insect body parts can be hardened by the addition of metals, and because niche separation may require morphological adaptations, inclusion of such metals may be linked to life history traits. Here, we analysed the distribution and enrichment of metals in the mandibles and ovipositors of a large family of gall-inducing wasps (Cynipidae, or Gall-Wasps) (plus one gall-inducing Chalcidoidea), and their associated wasps (gall-parasitoids and gall-inquilines) (Cynipidae, Chalcidoidea and Ichneumonoidea). Both plant types/organs where galls are induced, as well as galls themselves, vary considerably in hardness, thus making this group of wasps an ideal model to test if substrate hardness can predict metal enrichment. Non-galler, parasitic Cynipoidea attacking unconcealed hosts were used as ecological “outgroup”. With varying occurrence and concentration, Zn, Mn and Cu were detected in mandibles and ovipositors of the studied species. Zn tends be exclusively concentrated at the distal parts of the organs, while Mn and Cu showed a linear increase from the proximal to the distal parts of the organs. In general, we found that most of species having metal-enriched ovipositors (independently of metal type and concentration) were gall-invaders. Among gall-inducers, metals in the ovipositors were more likely to be found in species inducing galls in woody plants. Overall, a clear positive effect of substrate hardness on metal concentration was detected for all the three metals. Phylogenetic relationships among species, as suggested by the most recent estimates, seemed to have a weak role in explaining metal variation. On the other hand, no relationships were found between substrate hardness or gall-association type and concentration of metals in mandibles. We suggest that ecological pressures related to oviposition were sufficiently strong to drive changes in ovipositor elemental structure in these gall-associated Hymenoptera.

## Introduction

The stiffness, hardness and thickness of arthropod cuticle is extremely variable [1], [2], and in certain species and body parts can be reinforced by the addition of Zn, Mn or other elements [3], [4]. Metals and halogens have been found in the mandibles, chelicerae, stings, pedipalps, forcipules, leg claws and ovipositors, and typically at prone-to-wear cutting edges of these organs [3], [5], [6], [7], [8], [9]. The inclusion of such elements can greatly improve cuticle hardness. For example, removal of Zn from worm jaws decreases hardness by over 65% [10], and in ants [4] and termites [11] the hardness of the mandibular teeth correlates with Zn content. For other metals, such as Mn, quite common but found in minor concentrations, it is still not clear the effect on cuticle mechanical properties [11]. Cuticle enriched by Zn and Mn are believed to differ in their mechanical properties [5] and so their differential use may indicate different functional roles.

Because resource partitioning and niche separation may require special adaptations which often include morphological changes [12], [13], from an evolutionary point of view, inclusion of metals in the cuticle may be linked to life history traits. This hypothesis seems to be true in some studied cases. For example, the presence of harder mandibles in the drywood termites seems to be related to lack of access to free water with which to moisten wood, with Zn being rare or absent in termites able to moisten wood [14]; high concentrations of Zn and Mn were found in mandibles of insect larvae that bore into seeds, but not in mandibles of insect larvae that attack previously damaged seeds [15].

On the other side, there is some indication that the presence of metals in selected organs can be related to phylogenetic relationships, as in the case of mandibles of herbivorous insects: Mn is not found in the Orthoptera, Phasmatidae and Lepidoptera, while in Formicidae both Zn and Mn are present [16]. Within parasitic Hymenoptera (Parasitica), the cuticle of the ovipositor is sometimes reinforced by either Zn (Siricidae, Stephanidae) or Mn (Cynipoidea, Ichneumonoidea) or both (Megalyridae) [17], [6]. Quicke et al. [18] even reported Ca in the ovipositor tip of a few ichneumonoids. Thus, the strongest predictor of whether an organism contains metals may not be its behaviour or habitat, but whether or not other members of its family also use such elements [16], [19], [3], [7], [20].

Here, we analysed the distribution and concentration/enrichment of metals in the mandibles and ovipositors primarily of a large family of gall-inducing wasps (Hymenoptera: Cynipidae, or Gall-Wasps), and of some of their associated wasps (gall-parasitoids and gall-inquilines, altogether named here as gall-invaders) (Hymenoptera: Cynipidae, Chalcidoidea and Ichneumonoidea). Cynipidae, a species-rich family of gall-inducing and gall-inquiline wasps, with roughly 1400 described species, represents the second largest radiation of gall-inducing insects after gall midges (Diptera: Cecidomyiidae) [21], [22]. The gall-inducing cynipids form galls, morphological structures formed by plants in response to gall inducer organisms, inside which the larvae develop [23], [22]. Galls induced by Gall-Wasps are morphologically complex and provide shelter and nutrition for their larvae, as well as protection from predators and parasitoids [24], [25]. Notably species in the tribe Cynipini and few species in the tribe Pediaspidini have complex cyclically parthenogenetic (heterogonic) life cycles (i.e. alternation of sexual and asexual generations), which in some cases also involve host plant alternation (heteroecy) [26]. The cynipid inquilines also have phytophagous larvae but cannot initiate gall formation on their own. Instead, their larvae develop inside the galls induced by other Gall-Wasps [27]. On the other side, the Chalcidoidea and Ichneumonoidea associated with galls are mainly parasitoids of larvae of Cynipidae [28], [29], [30] and other insects (e.g. [31]). The genus *Aditrochus*, here studied, belongs to Chalcidoidea but induces galls.

Selected species of parasitic groups of Cynipoidea (Figitidae) phylogenetically related to Cynipidae, but not associated with galls, were also analysed. These selected figitid species are endoparasitoids of other insects not concealed in a substrate [32], and served here as a sort of “ecological outgroup”. The genus *Parnips*, also here studied, is unique among Figitidae, being a parasitoid in cynipid galls (it is thus a gall-invader).

Patterns of metal incorporation have been almost not investigated to date in Gall-Wasps and their associated wasps [6].

Here we tested for the hypothesis that metal incorporation is more likely to occur (and at higher concentrations) for wasp species ovipositing in harder substrates. This hypothesis includes two possible predictions. First, species primarily associated to galls as inquilines or parasitoids (gall-invaders), and thus ovipositing in galls, which are typically thick and often hard structures, may require a greater incorporation of metals in the ovipositors, compared with gall-inducing species, which oviposit in plant tissues, and with non-gall parasitoids. Second, because gall-inducers oviposit in plant types and organs differing in hardness (from herbs to trees, and from leaves and flowers to buds and roots), we expected a positive relationship between the hardness of plant substrate and ovipositor metal enrichment. In addition, we also tested the hypothesis that the hardness of the emerging substrate (i.e. the gall for most taxa of our sample, plus larval cuticle for non-gall parasitoids) positively correlates with mandible metal enrichment.

## Materials and Methods

### Selected Taxa for Study

Females of 43 species of Gall-Wasps of the eight described cynipid tribes and the main genera of Cynipidae, seven species of Chalcidoidea and Ichneumonidae (all but one acting as gall-parasitoids and one as gall-inducer), and six species of Figitidae (five not associated with galls and one acting as gall-parasitoid) were investigated (Table 1). For heterogonic species, either sexual or asexual forms (both forms for two species) were used. The studied gall-associated taxa were selected to represent, from one side, all the main lineages of gall-inducers (Cynipidae) spanning a wide range of biologies (e.g. plant type, gall structure) and, from the other side, the taxonomic and biological diversity of gall-invaders (inquilines and parasitoids) (Table 1). Both mandibles and ovipositors were not available for all 56 species: in particular, mandibles were not studied for 3 species and ovipositor was not studied for 6 species and the asexual form of one species. As in [6] we preferred to examine 1 or few individuals of closely related taxa with similar biology rather than many representatives of the same species. Overall, a total of 86 females (1.46±0.6 per species on average) were studied. Voucher specimens are deposited at Museo Nacional de Ciencias Naturales (CSIC) (Madrid, Spain).

**Table 1:** List of the studied species (with number of individuals in brackets), together with biological information and ranks for metal concentrations in mandibles and in ovipositors.

Taxonomicclassification	Species	Biology	Mandible			Ovipositor			Collection country
			Zn	Mn	Cu	Zn	Mn	Cu	
Cynipoidea: Cynipidae: Aylacini	*Aulacidea freesei* Nieves-Aldrey 1994 (2)	Galler on *Silybum*	3	0	0	0	0	0	Spain
Cynipoidea: Cynipidae: Aylacini	*Aulacidea tragopogonis* (Thomson 1877) (2)	Galler on *Tragopogon*	3	0	0	0	0	0	Spain
Cynipoidea: Cynipidae: Aylacini	*Aylax papaveris*(Perris 1839) (2)	Galler on *Papaver*	1	0	0	0	0	0	Spain
Cynipoidea: Cynipidae: Aylacini	*Diastrophus rubi* (Bouche 1834) (3)	Galler on *Rubus*	4	0	2	0	0	1	Spain
Cynipoidea: Cynipidae: Aylacini	*Hedickiana levantina* (Hedicke 1928) (1)	Galler on *Salvia*	3	1	4	0	0	0	Jordan
Cynipoidea: Cynipidae: Aylacini	*Iraella luteipes* (Thomson 1877) (2)	Galler on *Papaver*	1	0	0	0	0	0	Spain
Cynipoidea: Cynipidae: Aylacini	*Isocolus lichtensteini* (Mayr 1882) (2)	Galler on *Centaurea*	4	2	2	0	1	0	Spain
Cynipoidea: Cynipidae: Aylacini	*Liposthenes kerneri* (Wachtl 1891) (1)	Galler on *Nepeta*	4	1	2	0	0	0	Spain
Cynipoidea: Cynipidae: Aylacini	*Panteliella fedtschenkoi* (Rubsaamen 1896) (1)	Galler on *Phlomis*	3	2	3	0	0	0	Romania
Cynipoidea: Cynipidae: Aylacini	*Phanacis centaureae* Förster 1860 (1)	Galler on *Centaurea*	3	1	0	0	0	0	Spain
Cynipoidea: Cynipidae: Aylacini	*Timaspis phoenixopodos* Mayr 1882 (1)	Galler on *Lactuca*	2	0	0	0	0	0	Spain
Cynipoidea: Cynipidae: Aylacini	*Xestophanes potentillae* (Retzius in De Geer 1773) (1)	Galler on *Potentilla*	3	2	0	0	0	0	Spain
Cynipoidea: Cynipidae: Cynipini	*Andricus burgundus* Giraud 1859 (sexual) (3)	Galler on *Quercus*	2	0	0	0	0	0	Spain
Cynipoidea: Cynipidae: Cynipini	*Andricus coriarius* (Hartig 1843) (asexual) (2)	Galler on *Quercus*	4	0	2	0	2	0	Spain
Cynipoidea: Cynipidae: Cynipini	*Andricus crispator* Tschek 1871 (sexual) (2)	Galler on *Quercus*	3	0	0	–	–	–	Spain
Cynipoidea: Cynipidae: Cynipini	*Andricus curvator* Hartig 1840 (sexual) (2)	Galler on *Quercus*	2	0	0	0	0	0	Spain
Cynipoidea: Cynipidae: Cynipini	*Andricus grossulariae* Giraud 1859 (asexual) (3)	Galler on *Quercus*	3	0	0	0	0	0	Spain
Cynipoidea: Cynipidae: Cynipini	*Andricus grossulariae* Giraud 1859 (sexual) (2)	Galler on *Quercus*	4	0	4	0	2	0	Spain
Cynipoidea: Cynipidae: Cynipini	*Andricus multiplicatus* Giraud 1859 (sexual) (2)	Galler on *Quercus*	3	0	0	–	–	–	Hungary
Cynipoidea: Cynipidae: Cynipini	*Andricus pictus* (Hartig 1856) (asexual) (3)	Galler on *Quercus*	4	0	0	0	2	0	Spain
Cynipoidea: Cynipidae: Cynipini	*Andricus quercusradicis* (Fabricius 1798) (asexual) (2)	Galler on *Quercus*	3	0	0	–	–	–	Spain
Cynipoidea: Cynipidae: Cynipini	*Andricus quercusradicis* (Fabricius 1798) (sexual) (1)	Galler on *Quercus*	2	0	0	0	2	0	Spain
Cynipoidea: Cynipidae: Cynipini	*Andricus quercusramuli* (Linnaeus 1761) (sexual) (2)	Galler on *Quercus*	2	0	0	0	0	0	Spain
Cynipoidea: Cynipidae: Cynipini	*Biorhiza pallida* (Linnaeus 1758) (asexual) (1)	Galler on *Quercus*	1	0	1	0	4	2	Spain
Cynipoidea: Cynipidae: Cynipini	*Cynips quercusfolii* Linnaeus 1758 (asexual) (2)	Galler on *Quercus*	2	2	0	0	4	0	Spain
Cynipoidea: Cynipidae: Cynipini	*Dryocosmus kuriphilus* Yasumatsu 1951 (1)	Galler on *Castanea*	3	1	2	0	0	0	Italy
Cynipoidea: Cynipidae: Cynipini	*Plagiotrochus gallaeramulorum* (Fonscolombe 1832) (asexual) (1)	Galler on *Quercus*	3	1	2	–	–	–	Spain
Cynipoidea: Cynipidae: Cynipini	*Plagiotrochus quercusilicis*(Fabricius 1798) (sexual) (1)	Galler on *Quercus*	3	2	0	–	–	–	Spain
Cynipoidea: Cynipidae: Cynipini	*Pseudoneuroterus macropterus* (Hartig 1843) (asexual) (2)	Galler on *Quercus*	4	2	0	0	3	0	Hungary
Cynipoidea: Cynipidae: Cynipini	*Trigonaspis mendesi* Tavares 1902 (asexual) (1)	Galler on *Quercus*	3	1	0	0	0	0	Spain
Cynipoidea: Cynipidae: Cynipini	*Trigonaspis synaspis* (Hartig 1841) (sexual) (2)	Galler on *Quercus*	1	0	0	0	0	0	Spain
Cynipoidea: Cynipidae: Diplolepidini	*Diplolepis rosae* (Linnaeus 1758) (2)	Galler on *Rosa*	1	0	0	0	0	0	Spain
Cynipoidea: Cynipidae: Eschatocerini	*Eschatocerus acaciae* Mayr 1881 (2)	Galler on *Prosopis*	2	2	1	0	0	1	Argentina
Cynipoidea: Cynipidae: Paraulacini	*Cecinothofagus gallaelenga*Nieves-Aldrey & Liljeblad 2009 (1)	Gall-parasitoid or inquiline of *Aditrochus*	1	0	0	–	–	–	Chile
Cynipoidea: Cynipidae: Pediaspidini	*Pediaspis aceris* (Gmelin 1790) (asexual) (1)	Galler on *Acer*	1	1	1	0	0	0	Spain
Cynipoidea: Cynipidae: Qwaqwaiini	*Qwaqwaia scolopiae* Liljeblad, Nieves-Aldrey &Melika 2011 (1)	Galler on *Scolopia*	3	1	0	1	0	1	South Africa
Cynipoidea: Cynipidae: Synergini	*Ceroptres cerri* Mayr 1873 (1)	Gall-inquiline of *Plagiotrochus*+other Cynipini	2	0	1	0	2	1	Spain
Cynipoidea: Cynipidae: Synergini	*Periclistus brandtii* (Ratzeburg 1832) (1)	Gall-inquiline of *Diplolepis*	1	2	2	0	2	3	Spain
Cynipoidea: Cynipidae: Synergini	*Rhoophilus loewi* Mayr 1881 (1)	Gall-inquiline of *Scyrotis* (Lepidoptera: Cecidosidae)	3	1	2	0	0	1	South Africa
Cynipoidea: Cynipidae: Synergini	*Saphonecrus lusitanicus* (Tavares 1901) (1)	Gall-inquiline of *Andricus+Plagiotrochus*	1	1	0	0	2	1	Spain
Cynipoidea: Cynipidae: Synergini	*Synergus clandestinus* Weld 1952 (1)	Gall-inquiline of *Andricus*	4	2	3	2	3	2	Spain
Cynipoidea: Cynipidae: Synergini	*Synergus hayneanus* (Ratzeburg 1833) (1)	Gall-inquiline of *Andricus*	2	1	1	0	4	0	Spain
Cynipoidea: Cynipidae: Synergini	*Synergus physocerus* Hartig 1843 (1)	Gall-inquiline of *Andricus*	2	0	2	0	1	0	Spain
Cynipoidea: Cynipidae: Synergini	*Synergus umbraculus* (Olivier 1791) (2)	Gall-inquiline of *Andricus*	3	1	2	0	3	0	Spain
Cynipoidea: Cynipidae: Synergini	*Synophrus politus* Hartig 1843 (2)	Gall-inquiline of *Andricus*	3	1	0	0	2	0	Spain
Cynipoidea: Figitidae: Anacharitinae	*Acanthaegilips* sp. (1)	Endoparasitoid of Neuroptera: Chrysopidae and Hemerobiidae	3	0	1	–	–	–	Colombia
Cynipoidea: Figitidae: Aspicerinae	*Callaspidia notata* (Boyer de Fonscolombe 1832) (1)	Endoparasitoid of Diptera: Cyclorrhapha	3	1	2	0	0	0	Spain
Cynipoidea: Figitidae: Charipinae	*Apocharips* sp. (1)	Endoparasitoid of Hymenoptera: Braconidae and Chalcidoidea	–	–	–	0	0	0	Spain
Cynipoidea: Figitidae: Eucolinae	*Ganaspis* sp. (1)	Endoparasitoid of Diptera: Cyclorrhapha	2	1	1	0	0	0	Spain
Cynipoidea: Figitidae: Figitinae	*Neralsia* sp. (1)	Endoparasitoid of Diptera: Cyclorrhapha	2	1	1	0	0	2	Colombia
Cynipoidea: Figitidae: Parnipinae	*Parnips nigripes* (Barbotin 1963) (1)	Gall-parasitoid of cynipid galler in *Papaver*	4	1	2	0	1	0	Spain
Chalcidoidea: Eupelmidae	*Eupelmus spongipartus* Förster 1860 (1)	Gall-parasitoid of *Callirhytis*	1	0	1	0	1	0	Spain
Chalcidoidea: Ormyridae	*Ormyrus nitidulus* (Fabricius 1804) (1)	Gall-parasitoid of *Andricus*	4	3	3	4	0	1	Spain
Chalcidoidea: Pteromalidae	*Aditrochus fagicolus* Ruebsaamen 1902 (1)	Galler on *Nothophagus*	–	–	–	3	0	2	Chile
Chalcidoidea: Pteromalidae	*Pteromalus bedeguaris* (Thomson 1878) (1)	Gall-parasitoid of *Diplolepis*	1	1	0	1	0	1	Spain
Chalcidoidea: Torymidae	*Megastigmus stigmatizans* Fabricius 1798 (1)	Gall-parasitoid of *Andricus*	4	0	0	1	1	0	Spain
Chalcidoidea: Torymidae	*Torymus* sp. (1)	Gall-parasitoid of *Eschatocerus*	–	–	–	2	0	0	Argentina
Ichneumonoidea: Ichneumonidae	Ichneumonidae sp. (1)	Gall-parasitoid of *Biorhiza*	1	0	0	0	1	1	Spain

In the mandibles, Zn was ranked as 1 (<5 wt%), 2 (5.1–10 wt%), 3 (10.1–20 wt%) or 4 (>20.1 wt%), while the other metals were ranked as 1 (<0.3 wt %), 2 (0.31–0.6 wt%), 3 (0.61–2 wt%) or 4 (>2 wt%). In the ovipositor, all metals were ranked as 1 (<0.3%), 2 (0.31–0.6 wt%), 3 (0.61–2 wt%) or 4 (>2 wt%). “–” identifies that for that species the organ used to emerge (mandibles) or to oviposit (ovipositor) was not analysed.

For all species except two collected in Chile, no specific permissions were required for the locations/activities, since collections were done in non-protected areas. The two species from Chile were collected in the Reserva Nacional Los Queules, and the permit for such collection was issued by the Corporación Nacional Forestal (CONAF). The field studies did not involve endangered or protected species.

### Scanning Electron Microscopy (SEM) and Energy Dispersive X-ray Spectroscopy (EDS)

The micromorphology, topography, distribution and detection of metals were determined using a Philips FEI INSPECT (Hillsboro, Oregon, USA), a scanning electron microscope (SEM) at the Museo Nacional de Ciencias Naturales (CSIC). To obtain comparative results, we always worked in a high-vacuum mode with a backscattered electron detector (BSED) under vacuum conditions of 30 Pa, a high voltage of 20 kV, a suitable beam spot diameter for particular magnifications and to achieve good focus and astigmatism correction, and a working distance of approximately 10 mm to the detector. The X-ray energy microanalysis (EDS) of the samples and the analysis for line-scan were conducted with an energy-dispersive X-ray spectrometer (INCA Energy 200 energy dispersive system, Oxford Instruments), as it was previously done in similar works [15], [33].

Females were dissected under light microscopy and the excised ovipositors and mandibles were gold-coated after mounting on adhesive carbon pads attached to aluminum stubs. The hymenopteran ovipositor proper consists of three valves, with the upper valve and a pair of lower valves; these together form the egg canal [34]. The ovipositor sheaths (i.e. the third valvae primarily serving for protecting the ovipositor proper, [34]) were not considered in this study since they do not enter substrate during oviposition. For the few specimens coming from the Museum collection, we introduce in the SEM the whole, not gold-coated, individuals.

The semi-quantitative analysis allowed us to establish not only which elements were present but also the concentration of each element, which required an accurate intensity measurement for each peak in the spectrum [35]. We used the maximum peak intensities obtained by a least-squares fitting routine that used standard peaks correlated to a spectrum of known compounds [36]. After these intensities were determined, matrix corrections were applied [37] to determine the concentration of each element. This correction method uses approximated exponential curves and the φ(ρZ) model to describe the shape of the curves. Thus, improved measurements of light elements in a heavy-element-rich matrix and samples that are tilted in the direction of the incident electron beam can be obtained [37].

The correction factors are dependent on the sample composition (which is the object of our analysis), so that the actual concentrations must be derived using an iterative procedure [38]. Apparent concentrations are then used to calculate correction factors and make more “accurate” estimates of the concentrations. After successive iterations, concentrations that are accurate to approximately 0.01% can be achieved [38].

To calculate the statistical error in the concentration, the weight percentage of the sigma value should be used to determine whether the element is below the detection limits of the sample analysis. We were conservative in this study and used a stricter condition that requires an element’s weight percentage to be greater than three times the weight percentage of the sigma value resulting from the analysis [39].

The Smart Map application was used to collect and store X-ray for production of line-scans and quantification. The analytical conditions for 0–20 keV spectral range were optimized for the best average spectra: process time of 5, resolution of 128 × 112 pixels, dwell time of 6000 µs and live acquisition time of 300 s.

For each specimen, we first performed a point-analysis, in which metal concentration was obtained at one point on the distal part of the organ (mandible tooth point and ovipositor distal point) and on the inner part of the organ (i.e. more basal position) (Fig. S1). Then, we performed a line-scan analysis to study the metal concentration along a line starting from the distal point of the point-analysis and ending at about 50–800 µm (depending on the size of the organ and its position) in the inner side (Fig. S1). In the line-scan analysis, the concentrations are calculated averaging the values recorded across the line, thus giving an overall metal enrichment in the cuticle. In addition, from the rough data of the line-scan it was possible to analyse the dispersion pattern across the line (distance). In such a way, we could estimate if the metal concentration changes across the line (e.g. if increase from inner to outer point, the distal cutting/drilling part of the organ) and with which shape.

Since the analytical method of metal detection is semi-quantitative, we ranked all the obtained values [6]. In the mandibles, Zn, which can be very abundant (see results) was ranked as 1 (<5 wt%), 2 (5.1–10 wt%), 3 (10.1–20 wt%) or 4 (>20.1 wt%); in the ovipositor, Zn was much less abundant and was ranked as 1 (<0.3%), 2 (0.31–0.6 wt%), 3 (0.61–2 wt%) or 4 (>2 wt%). In both mandibles and ovipositors, Mn and Cu were ranked as Zn in the ovipositor. We ranked both the concentrations recorded with the distal point analysis and with the line-scan. If significant patterns of increase in the line-scan were detected and resulted in a higher rank at the distal point than the rank of the average concentration observed with the line-scan, we used in the statistical analysis the former rank (distal point).

### Statistical Analysis

Despite the few individuals studied per species limit conclusions about intra-specific variability, an exploration of the ranked values of metals in the studied specimens strongly suggests this variability is small. In fact, the recorded values of metal concentrations always fall in the same ranks for individuals belonging to the same species. Thus, species were treated as single points in the statistical analysis and individuals were not considered. Separate analyses were performed for mandibles and ovipositors.

To study if metal concentrations increase from the inner to the distal part of the organs, the curves obtained by the line-scan method were fitted to either a linear model or a sigmoid model, with model significance tested with linear or non-linear regressions. We reported all the significant regressions (P<0.05), but because the large sample size in these analysis (i.e. the points along the line-scan, n >100) tended to give significant regressions at low R^2^ [40], we also evidenced in particular those in which the distance from the tip of the organ explains at least half of the variance in metal concentration (R^2^≥0.50) (Table S2).

To explore the dissimilarity among species based on the concentration of the different metals, we performed a hierarchical cluster analysis, which finds relatively homogeneous clusters of cases based on measured characteristics (in this study rank values of metal concentrations) [41]. The cluster analysis was performed through Ward’s method based on Euclidean distance (dissimilarity) between pairs of objects. This analysis also reported the dissimilarity value (truncation), which likely determines how many clusters best suit the data.

The ordinal nature of our independent variables (metal concentration ranks) did not allow to apply classic binary logistic regressions and standard linear regressions to test for association between substrate hardness and metal concentration. Thus, we used appropriate statistics for ordinal and binary variables.

Spearman's rank correlation test was used to look for associations between the concentrations (ranks) of the different metals across species.

To study if metal enrichment is linked to species ecology (substrate hardness), we performed ordinal regression analysis (probit model), commonly used for predicting an ordinal variable [42]. As with classical logistic regression, this models estimate a chi-square (i.e., likelihood ratio) which compares deviances for the full model to the deviance for the baseline or null model. For both organs, one model per each metal was carried out.

Substrate hardness refers to different concepts directly related to emerging or ovipositing. For the mandibles, the substrate to be dug during adult emergence is the gall or, in case of non-gall parasitoids, the host body. This emerging substrate was ranked as 1 (host larval cuticle), 2 (soft-juicy galls), 3 (dry-hard galls without woody external layer) and 4 (very hard galls with woody external layer). For the ovipositor, the substrate to be drilled during egg-laying is the plant tissue (for gall-inducers), the galls (for gall-invaders), or the larval host body (for non-gall parasitoids). This oviposition substrate was ranked as 1 (host larval cuticle), 2 (mostly herbaceous plants), 3 (mostly woody plants and relatively soft/immature galls) and 4 (hard mature galls). Such ranking for substrate hardness was based on previously published information on the biology of the studies species (mainly [43] and references therein cited) (see Table S1).

### Trait Mapping on Phylogeny

To map our results on a phylogeny of the studied species, we draw an intuitive “handmade” phylogenetic tree based on combined molecular and morphological phylogenetic analysis available in recent works [44], [45], [46], [47], [48], [49], [50] and more recent unpublished results obtained in an on-going study in which one of the authors of the present paper (JLN-A) is involved. Relationships among the studied species of Gall-Wasps (Cynipidae) were mostly based on [44], which performed an analysis based on three molecular markers; that study included all the tribes except the small Qwaqwaiini and Paraulacini, in our study represented by one species each. The phylogenetic position of *Cecinothofagus gallaelenga* (Paraulacini: basal to Cynipini+Synergini+Aylacini+Qwaqwaiini) was inferred after [48] and unpublished results. For the phylogenetic position of *Qwaqwaia scolopiae* (Qwaqwaiini: basal to Cynipini) we referred to unpublished evidences. Additional information for some genera and species of Cynipini and Synergini (Cynipidae) not included in [44] was retrieved from posterior published molecular analyses [46], [47]. The phylogenetic position of the parasitic groups of Cynipoidea (Figitidae) was derived from recent combined molecular+morphological studies [44], [45], and the position on the tree of the Chalcidoidea and Ichneumonidae was also based on recent published results [49], [50]. It should be noted that, despite most of the depicted relationships are based on congruent results obtained with morphology and genetics (all the relationships among superfamilies and within Figitidae and Chalcidoidea), some relationships within Cynipidae look different when using morphological evidence only (discussed in [44]). In particular, Synergini appear to be monophyletic in the morphological analysis and polyphyletic in the molecular analysis (as we here draw). In addition, the morphological evidence suggests a basal position of herb-gallers (Aylacini) over wood-gallers, rather than placing non-*Quercus* wood-gallers (Diplolepidini, Eschatocerini and Pediaspidini) as the more ancestral tribes (as we here draw following the molecular analysis). On the other side, the Aylacini appear polyphyletic or paraphyletic in both analyses. Thus, the built tree could not be used to correct directly our results for common ancestry, but it was useful to roughly appreciate the relationships between phylogeny, metal occurrences and life-history traits, also taking into account the possible alternative tree typologies.

To quantitatively reinforce the visually suggested relationships among metal concentrations, substrate hardness and phylogeny, for ovipositor only (since no significant models were detected for mandibles, see results), we performed first the following comparisons of metal ranks (Mann-Whitney test): gall-invaders × gall-inducers (within Cynipidae), gall-invaders (within Cynipidae) × gall-invaders (outside Cynipidae), and non-gall parasitoids (thus outside Cynipidae) × gall-inducers (all groups). If the effect of phylogeny is weak, we expect larger differences in the first comparison than in all the other comparisons. Second, we performed the following comparisons of metal ranks: gall-inducers in substrate ranked 2 × gall-inducers in substrate ranked 3 (within Cynipidae) and gall-invaders in substrate ranked 3 × gall-inducers in substrate ranked 3 (all groups) (we used such hardness ranks because of the greater sample size). If the effect of phylogeny is weak, we expect larger differences in the first comparison than in the second one.

## Results

### Mandibles

**Figure 1:**
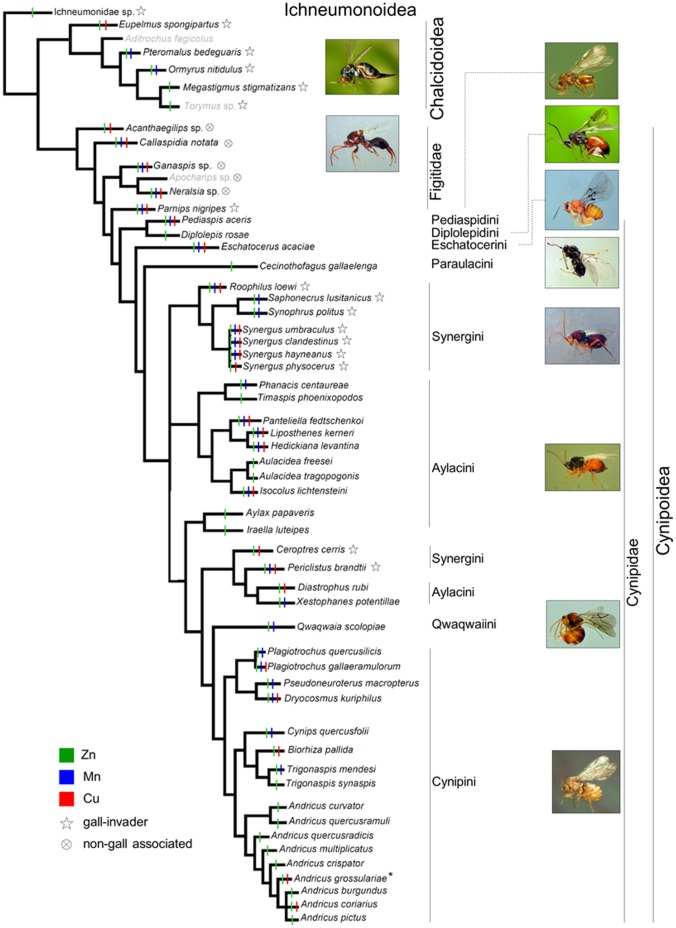
Occurrence of metals in the mandibles of the studied species, mapped on a phylogenetic tree derived from recent literature and unpublished data (see Methods). Species for which mandibles were not studied have their name in grey. * Zn and Cu are present in the sexual form, while only Zn is present in the asexual form.

**Figure 2:**
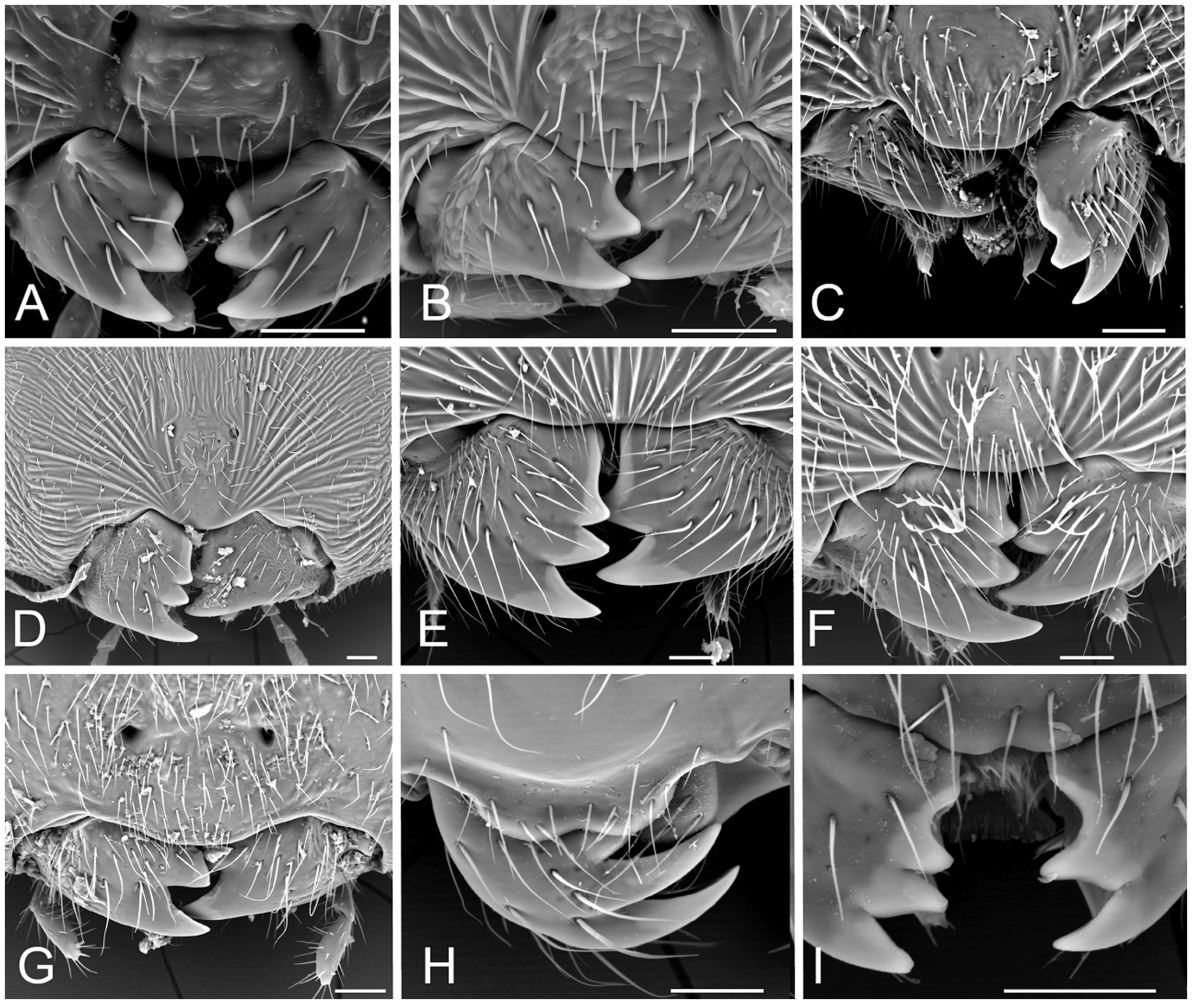
SEM pictures of the mandibles of some representative species studied. Note the whiter outer areas of the tooth, which correspond to Zn-enriched areas. A) *Andricus burgundus* (sex), B) *Plagiotrochus quercusilicis* (sex), C) *Timaspis phoenixopodos*, D) *Hedickiana levantina*, E) *Synergus umbraculus*, F) *Periclistus brandtii*, G) *Diplolepis rosae*, H) *Acanthaegilips* sp., I) *Ganaspis* sp. Bar: 50 µm.

We found Zn, Mn and Cu in mandibles (Table 1). Zn was present in all species (Fig. 1) with generally high concentrations, with only 12 species out of 55 showing Zn falling in the lowest rank (<5 wt%) and more than half of the species (31) showing Zn >10.1 wt% (ranks 3–4) (Table 1). The abundant Zn is even visible from SEM images of mandibles, in which a clearly whiter area is recognizable at the outer margins of the teeth (Fig. 2). Such pattern is confirmed by the elemental analysis, which invariably showed null values at the inner point and a sigmoid increase from the inner to the outer part of the teeth (increasing the rank) (Fig. S2A and Table S2). Mn was found in about half of the species (28) (Fig. 1), with concentration mainly (18 spp.) falling in lowest rank 1 (<0.3 wt%) (Table 1). Mn was found to increase in concentration from the inner to the outer part of the mandible teeth in about half of the cases; when this occurs, it increased following a linear trend (Fig. S2B and Table S2). Cu occurred in 26 species (Fig. 1) with concentration in most cases (21 spp.) below 0.6 wt% (ranks 1–2) (Table 1), and increased (linearly) in concentration towards the tip of the teeth in all these species (Fig. S2C and Table S2). For both Mn and Cu, the concentration at the inner point was null or extremely low and not higher than the error (see methods), while their concentration measured both at the distal point and from the line-scan fall in the same rank.

Across species, higher concentrations of Zn were associated with higher concentrations of Cu (Spearman’s ρ = 0.35, n = 55, P = 0.009), and higher concentrations of Cu were associated with higher concentrations of Mn (Spearman’s ρ = 0.36, n = 55, P = 0.007).

The cluster analysis depicted a dendrogram in which three main groups can be separated (Fig. S3A). The group more dissimilar to the other ones (group 1) included mostly (10 out of 15) species of gall-inducing wasps. The other two groups, much more similar one with the other, included each about half of the gall-invading species, mixed with gall-inducing and non-gall species. Thus, it seems that the gall-association type does not account for species grouping. This was confirmed by the probit model analysis, since no regressions of the metal ranks against substrate hardness were significant (Table 2).

**Table 2:** Logistic ordinal models (probit, stepwise (forward)) showing the effect of substrate hardness on the metal concentration ranks found in the mandibles and ovipositors of the studied species.

Organ	Metal	Goodness of fit	Wald
Mandibles	Zn	R^2^ = 0.098, df = 51	?^2^ = 0.01, df = 1, P = 0.91
	Mn	R^2^ = 0.138, df = 51	?^2^ = 0.74, df = 1, P = 0.39
	Cu	R^2^ = 0.128, df = 50	?^2^ = 0.03, df = 1, P = 0.86
Ovipositor	Zn	R^2^ = 0.44, df = 46	?^2^ = 9.76, df = 1, P = 0.002
	Mn	R^2^ = 0.19, df = 46	?^2^ = 8.47, df = 1, P = 0.004
	Cu	R^2^ = 0.23, df = 47	?^2^ = 6.49, df = 1, P = 0.029

### Ovipositors

**Figure 3:**
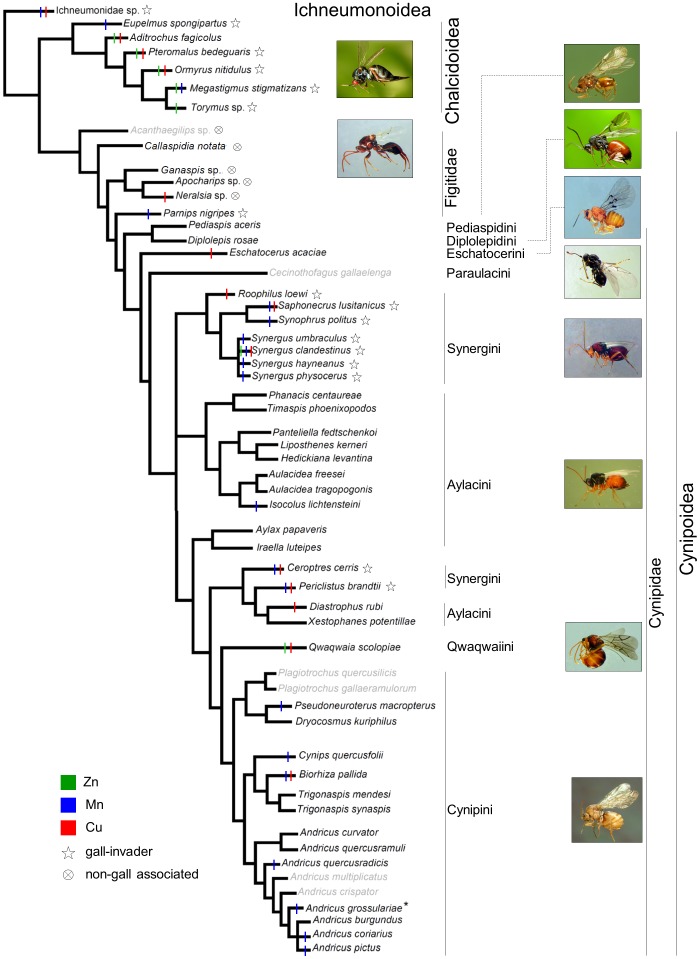
Occurrence of metals in the ovipositor of the studied species, mapped on a phylogenetic tree derived from recent literature and unpublished data (see Methods). Species for which the ovipositor was not studied have their name in grey (for *A. quercusradicis* data were available only for the sexual form). * Mn is present in the sexual form, while no metals were detected in the asexual form.

**Figure 4:**
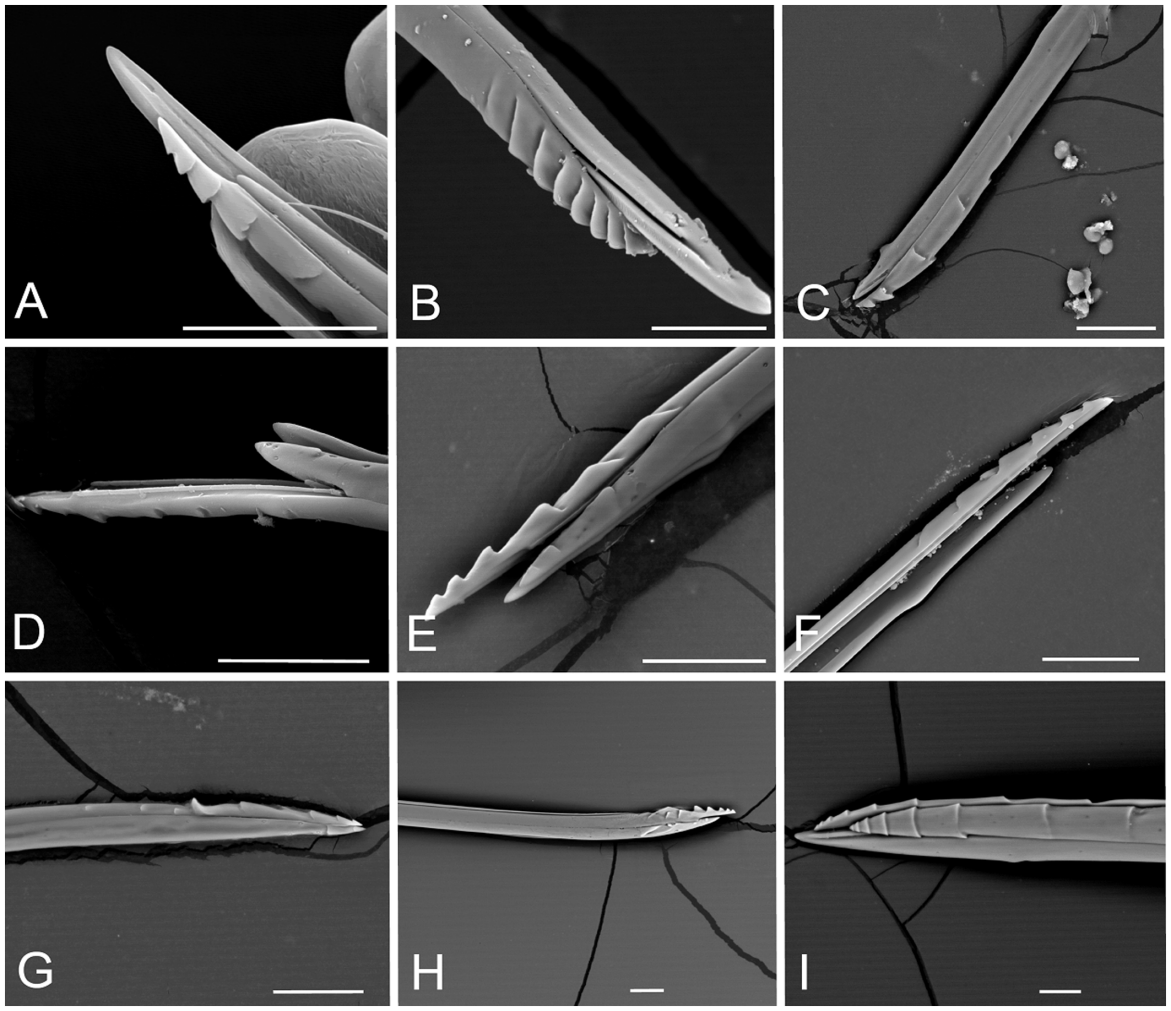
SEM pictures of the ovipositor of some representative species studied. A) Andricus quercusradicis (sex), B) Cynips quercusfolii (asex), C) Diastrophus rubi, D) Xestophanes potentillae, E) Synergus umbraculus, F) Synophrus politus, G) Callaspidia notata, H) Ormyrus nitidulus, I) Ichenumonidae sp. Bar: 50 µm.

We found Zn, Mn and Cu in the ovipositor, with different occurrence and concentration (Figs 3, Table 1). Metals were only found in the lower valvae (Fig. 4). Zn was present in only 7 species, all but two gall-invaders (Fig. 3), mainly at low concentrations (<0.3 wt%, 4 species) (Table 1). In Chalcidoidea only (four species), Zn was concentrated at the tip of the ovipositor compared to its inner part, following a sigmoid pattern of increase (increasing the rank) (Fig. S2E and Table S2). Mn was found in 20 species (Fig. 3), with concentration mainly (14 spp.) >0.6 wt% (ranks 2–4) (Table 1). Mn was found to increase in concentration from the inner to the outer part of the ovipositor, in linear fashion but not increasing the rank, in 14 of these species (11 of them Cynipidae) (Fig. S2F and Table S2). Cu occurred in 14 species (Fig. 3) with concentration in most cases (9 spp.) below 0.3 wt% (rank 1) (Table 1), and increased linearly towards the tip of the organ in five species, four of them gall-invaders (Fig. S2G and Table S2).

Across species, higher concentrations of Zn were associated with higher concentrations of Cu (Spearman’s ρ = 0.40, n = 52, P = 0.003).

The cluster analysis depicted a dendrogram in which three main groups can be separated (Fig. S3B). The more distant group (group 1) included most of the gall-invading species (12 out of 16). Group 2 included many gall-inducers and all the non-gall parasitoids, while group 3 was composed of two species, one gall-invader and one gall-inducer both in the Chalcidoidea.

In the probit model analysis, Zn, Mn and Cu ranks all increased with oviposition substrate hardness (Table 2). The effect of common ancestry on the observed variation seems to be weak. First, the differences in metal ranks between gall-invaders and gall-inducers within Cynipidae were significant, but they were not significant in the other contrasts (Table S3). Second, within Cynipidae, gall-inducers in substrate ranked 2 have less Zn and Mn than gall-inducers in substrate ranked 3, and that gall-invaders and gall-inducers in substrate ranked 3 (all groups) did not differ in Zn, Mn and Cu concentrations (Table S3). Several observations on the map of metal occurrence across the phylogenetic tree also suggest an overall weak role of common ancestry on the observed variability (see Discussion).

## Discussion

The present study is the first one dealing in detail with the occurrence and concentration of metals in the mandibles and ovipositors across a large sample of a species-rich superfamily of Hymenoptera (Cynipidae) in which members markedly vary in important life-history traits (from wood-gallers, to herb-gallers, to gall-invading inquilines) [23], [22], [51]. In addition, the inclusion in the analysis, on one side, of species of non-gall cynipoid parasitoids and, on the other side, of gall-invading wasps belonging to more distant hymenopteran groups (Chalcidoidea and Ichneumonoidea), made possible to test the hypothesis that life-history traits selected for the evolution of metal incorporation. We showed that the effect of greater oviposition substrate hardness significantly accounts for variability in metal inclusion in the ovipositor of gall-associated Hymenoptera, and that, overall, phylogenetic relationships probably have, even taking into account the few alternative tree typologies (see below), a weak effect on such variability. This would partially contrast with the view that occurrence of metals is mainly dependent on shared ancestry [20], in particular when looking at large (across families or orders) scale [6], [20]. The very few studies carried out within families, however, showed contrasting results. For example, Zn and Mn enrichment have been found in the mandibles of species of Formicidae which range widely in both habitat and in feeding behaviour [16], [3]; on the other side, the ability to moisten wood predicts the presence of Zn in termites lineages [14]. In Hymenoptera other than ants, the effect of phylogeny on metal enrichment within families was not clear to date, because the large survey of Quicke et al. [6] included many families but very few species per family.

We have interestingly shown that within-family variation in metal enrichment pattern occurs in Hymenoptera and relates with ecological traits. However, this seems to be true for the ovipositor only, since no links between life-history traits and variability of metal inclusion were observed in the mandibles of our sample. For example, Zn was present in the mandibles of all of our studies species, as well as in all the other hymenopterans analysed to date, with the exception of two aculeate wasps, two symphytan wasps, and only one member of Parasitica (Proctotrupoidea) [6]. Looking at the most recent superfamily-level phylogeny of Hymenoptera [48], it seems that Zn-enrichment appeared once in Symphyta, when the so-called Unicalcarida separated from the rest of the group, and then was conserved, in Apocrita, across all groups with only two apparent exceptions: one in the Pelecinidae (Parasitica: Proctotrupoidea) and one in Vespidae (Aculeata: Vespoidea).

Mn, on the other hand, is more rarely found in mandibles and it seems to have appeared twice within the so-called Proctotrupomorpha (within Parasitica): once in Cynipoidea and once in Chalcidoidea ([6], [48], this study). Within these groups, however, Mn may have been lost in some lineages, as in the genus *Andricus*.

Cu was detected in the mandibles of about half of the species, though with generally low concentrations. This result is interesting because Cu was very rarely reported in insects and in no hymenopterans to date. Adult mandibles of two termite species also contain Cu [14], [52]. Cu was found in internal organs of fruit flies, but not in the cuticle [53].

An association between metal inclusion and life-history traits seemed clearer for the ovipositor. Other aspects of hymenopteran ovipositor structure and morphology were already shown to be under selection imposed by ecological pressure [34], [54], [55], [56]. For example, the extent of sclerotisation of the ovipositor tip in fig wasps matched the force required to penetrate the syconium at the time of oviposition of each species [57]. Within Cynipoidea, all Figitinae and Eucoilinae that attack semi-concealed dipterous hosts were found to possess the so-called ovipositor clip (an adaptation for gripping host larvae during oviposition), while figitids that attack fully concealed hosts all lacked it [58]. Concerning metals, Quicke et al. [6] suggest at least seven independent acquisitions within the order, and within at least some superfamilies (e.g. Chalcidoidea, Ichneumonoidea) metal occurrence may correlate broadly with the way the ovipositor is used and the nature of the oviposition substrate. Thus, Zn is present in the ovipositor of species drilling deep in wood and not in related non-drilling species (e.g. siricids vs. xiphidriids within Symphita). Similarly, no metals were found in ovipositors of taxa that do not use their ovipositors to drill through any substrate [6]. However, some exceptions appeared: for example, the pimpline wasp, *Dolichomitus*, attack hosts that are concealed in hard wood, but no metal was found in its ovipositor [6], though this may be due to the fact that such wasps insert the ovipositor through pre-existing tunnels in the wood [59].

Here, we showed that drilling in galls, rather than in “normal” vegetation tissues or in unconcealed host larvae, also favoured metal-inclusion in the ovipositor of Hymenoptera. Because, at large scale, the distribution of gall-invading behaviour in the alternative phylogenetic trees does not suffer great variations, the overall association between metal inclusion and this life-history seems to be effectively probable. For example, distant groups as parasitoid Chalcidoidea and inquiline Synergini (Cynipoidea) have more metals, while in the non-gall Cynipoidea metals are basically absent. Considering separately the three studied metals the picture seems to be still valid, though with some exceptions which could suggest certain effects of phylogeny within families. For example, most of species having Zn and/or Cu in the ovipositor are gall-invaders (spanning three superfamilies), but it is also true that both gall-invading and gall-inducing pteromalids have these metals. On the other side, among gall-inducers, Zn and Cu were almost exclusively associated with harder substrates, supporting our hypothesis.

Mn was rarely found in association with Zn (two cases here, and one species of Megalyridae observed by [6]) and in several cases it was found in association with Cu. Though the role of Mn in hardening the insect cuticle is still debated [11], this metal could contribute to help drilling or cutting the substrate, in particular in such cases in which the organ follows preexisting gaps and cracks [6]. In the study of Quicke et al. [6], all the hymenopteran taxa having Mn-enriched ovipositor tips attack hosts concealed within a hard substrate, supporting this hypothesis. However, only some taxa analysed by Quicke et al. [6] attacking concealed hosts have metal hardened ovipositors; in addition, in our study Mn was also present in some gall-inducers, in particular within Cynipini, which have, however, to drill through woody (thus harder) substrates compared with herbs (only one herb-galler had Mn). As a support to the hypothesis that Mn inclusion co-evolved with substrate hardness, our results suggest that the observed variability in Mn weakly depend on phylogeny even within families. This is supported in particular by two observations and possibly an additional one (depending on the phylogenetic tree taken into account) First, *Parnips nigripes*, the only gall-invading figitid, is also the only figitid in our sample to have Mn. Second, a look at the tree built here [48] shows that Aylacini (herb-gallers) is composed of two different lineages in practice lacking Mn, with one of them more closely related to wood-galler cynipids (having Mn in various species). The morphology-based relationships, in which the Aylacini would be basal to wood-gallers within Cynipidae, would not alter the conclusion that an effect of substrate hardness exists, since in both phylogenetic scenarios herb-gallers would have lost metals previously acquired by either gall-invaders or wood-gallers. The third observation which may support a link between substrate hardness and metal inclusion concerns the inquiline Gall-Wasps (Synergini), but only if the relationships depicted by the three-genes molecular analysis are considered as the most probable. In fact, following this phylogenetic hypothesis, the Synergini are polyphyletic (including two lineages, respectively closer to the two groups of herb-gallers) and would have acquired metals twice independently (with Mn present in all except one case). However, following the sole morphological evidence, Synergini is a monophyletic group, thus not allowing hypotheses about independent associations between inquilinism and metal inclusion in the ovipositor.

### Conclusions

Overall, the mandibles and ovipositors of Gall-Wasps and gall-associated Hymenoptera are variably characterized by metal inclusion. While for mandibles such trait does not seem to have evolved with particular ecological pressures (emerging substrate hardness), for the ovipositor such relationship was clearer. First, the presence of Zn, Mn, Cu or their variable combination seems to be more likely to occur in species which penetrate galls; second, the hardness of substrate may have affected the evolution of metal allocation patterns in order to optimize such an “arm” directly linked with reproductive success. In particular, higher Mn levels would be basically associated with both woody (harder) vegetation substrate and gall-substrate (since it was most common among Cynipini and gall-invaders). Further studies devoted to investigate patterns of metal occurrence in the still unexplored groups of Hymenoptera (e.g. most Aculeata), together with a robust and large phylogeny covering all major subfamilies/tribes within the order, would greatly help to understand the evolutionary history and the adaptive significance of metal enrichment.

## Supporting Information

Figure S1 **SEM picture of a mandible (** ***Qwaqwaia scolopiae*** **) and of an ovipositor (** ***Iraella luteipes*** **), showing the inner (continuous white arrow) and distal (dashed white arrow) points used for the point analysis, and the line (black dashed) across which the line-scan analysis was performed.**
(TIF)

Figure S2 **Representative examples of the variation of metal concentration along the line-scan.** a, Zn (mandible); b, Mn (mandible); c, Cu (mandible); d, Zn (ovipositor); e, Mn (ovipositor); f, Cu (ovipositor). Trend lines are shown only for the significant regressions.
(TIF)

Figure S3 **Dendrograms depicted by the cluster analyses (Ward method) based on the concentration ranks of the different metals recorded for each species.** a, mandibles; b, ovipositor. The main groups recognized by the analysis are shown (the dissimilarity value which likely determines how many clusters best suit the data corresponds to the dashed line). Species whose names are in violet identify gall-invaders. For heterogonic species, (A) indicates the asexual form and (S) the sexual form.
(TIF)

Table S1 **Details of gall/substrate for each of the studied species, its rank, and references for hardness ranking.** Species are listed in alphabetic order. Gall description in the “Emerging site” column refers to the mature gall. “–” identifies that for that species the organ used to emerge (mandibles) or to oviposit (ovipositor) was not analysed.
(DOC)

Table S2 **Trends from outer to inner part of the organs as obtained after line-scan.** Species are listed in alphabetic order. Only significant models at P<0.05 are reported (in bold if R^2^≥0.5). “–”: organ not analyzed. Empty cell: no metals found or non-significant regressions.
(DOC)

Table S3 **Mann-Whitney contrasts performed to roughly correct the results of the logistic ordinal regressions for phylogenetic relationships among species.** In brackets, close to each metal tested, there are the compared medians.
(DOC)
