# Supplementary figures and images for: Breaking up the Wall: Metal-Enrichment in Ovipositors, but Not in Mandibles, Co-Varies with Substrate Hardness in Gall-Wasps and Their Associates
Source: PLoS One. 2013 Jul 24;8(7):e70529. doi: 10.1371/journal.pone.0070529 (PMC3722128; doi:10.1371/journal.pone.0070529)

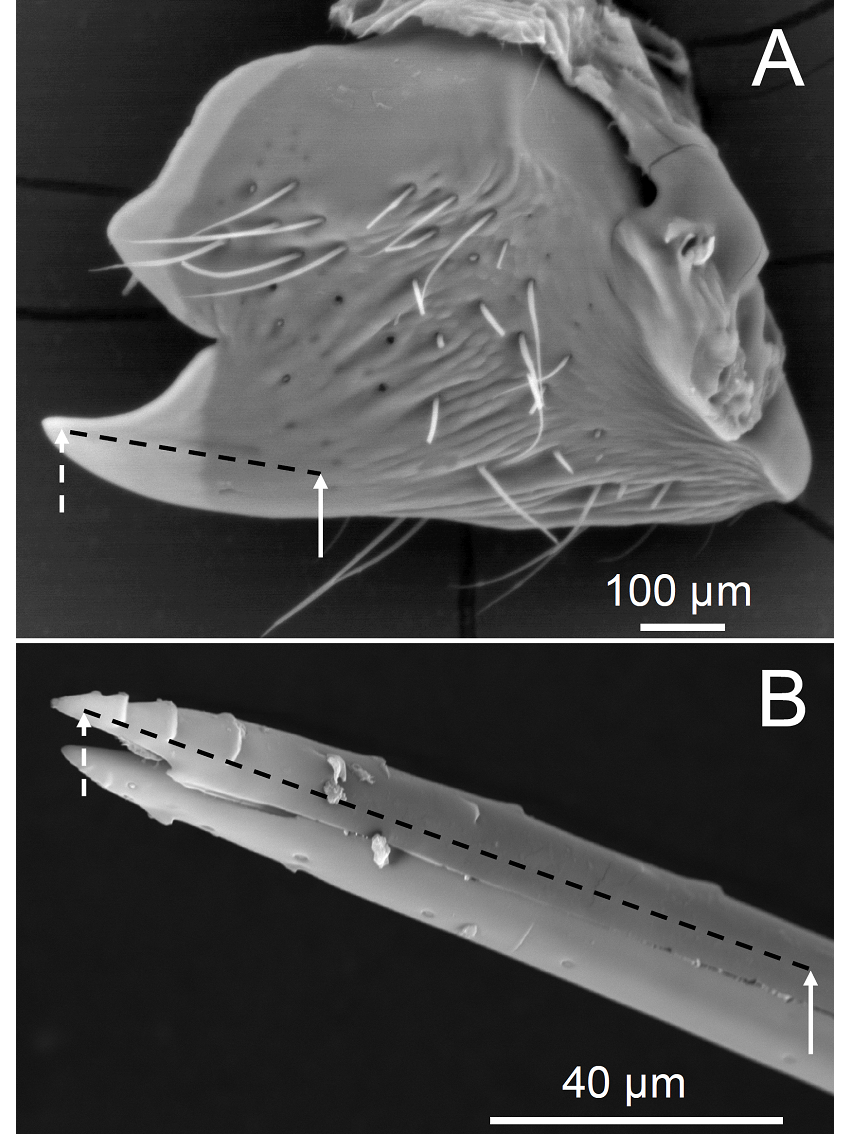

Supplement: Figure S1 — SEM picture of a mandible ( Qwaqwaia scolopiae ) and of an ovipositor ( Iraella luteipes ), showing the inner (continuous white arrow) and distal (dashed white arrow) points used for the point analysis, and the line (black dashed) across which the line-scan analysis was performed. (TIF) [file pone.0070529.s001.tif]

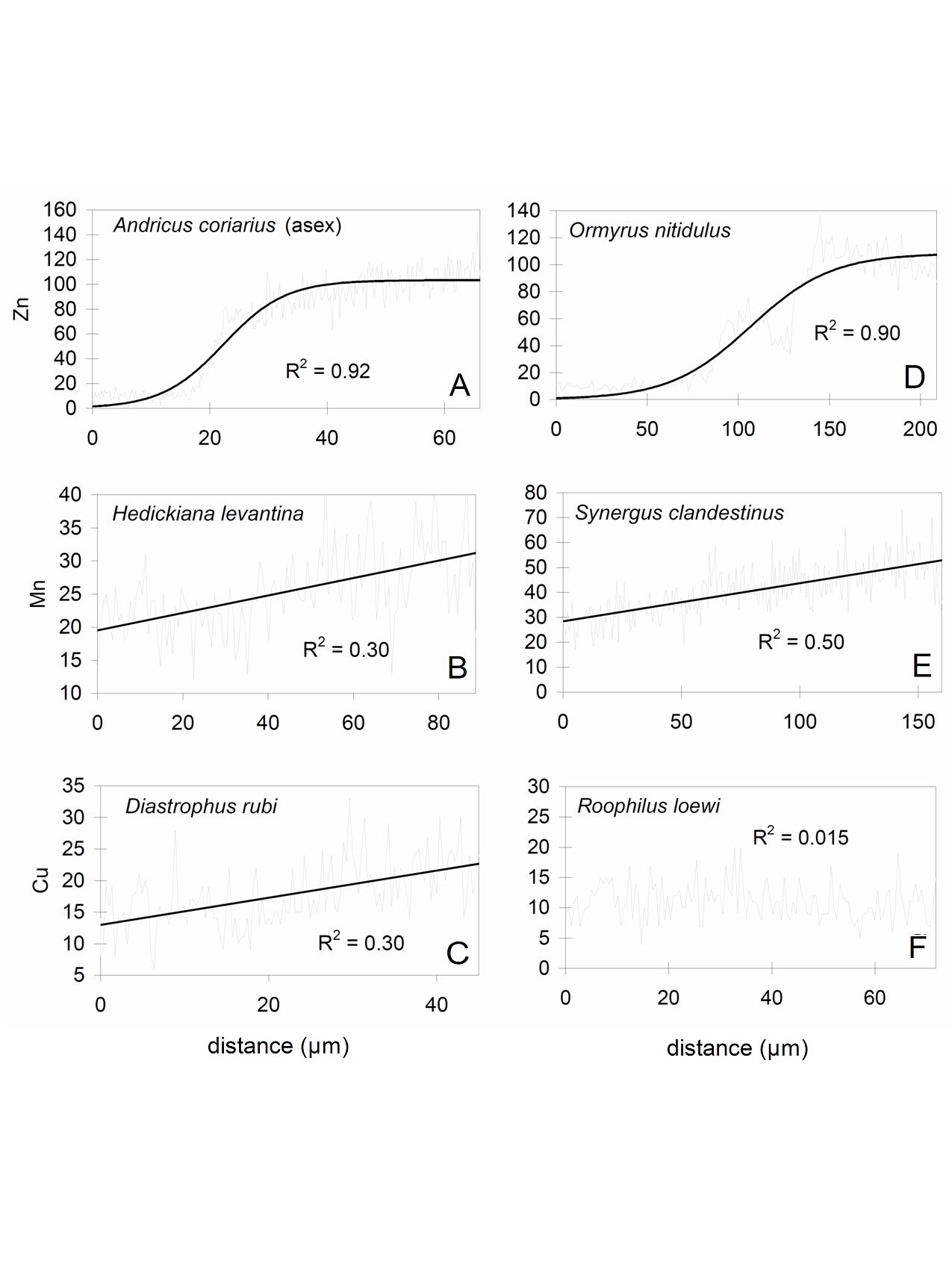

Supplement: Figure S2 — Representative examples of the variation of metal concentration along the line-scan. a, Zn (mandible); b, Mn (mandible); c, Cu (mandible); d, Zn (ovipositor); e, Mn (ovipositor); f, Cu (ovipositor). Trend lines are shown only for the significant regressions. (TIF) [file pone.0070529.s002.tif]

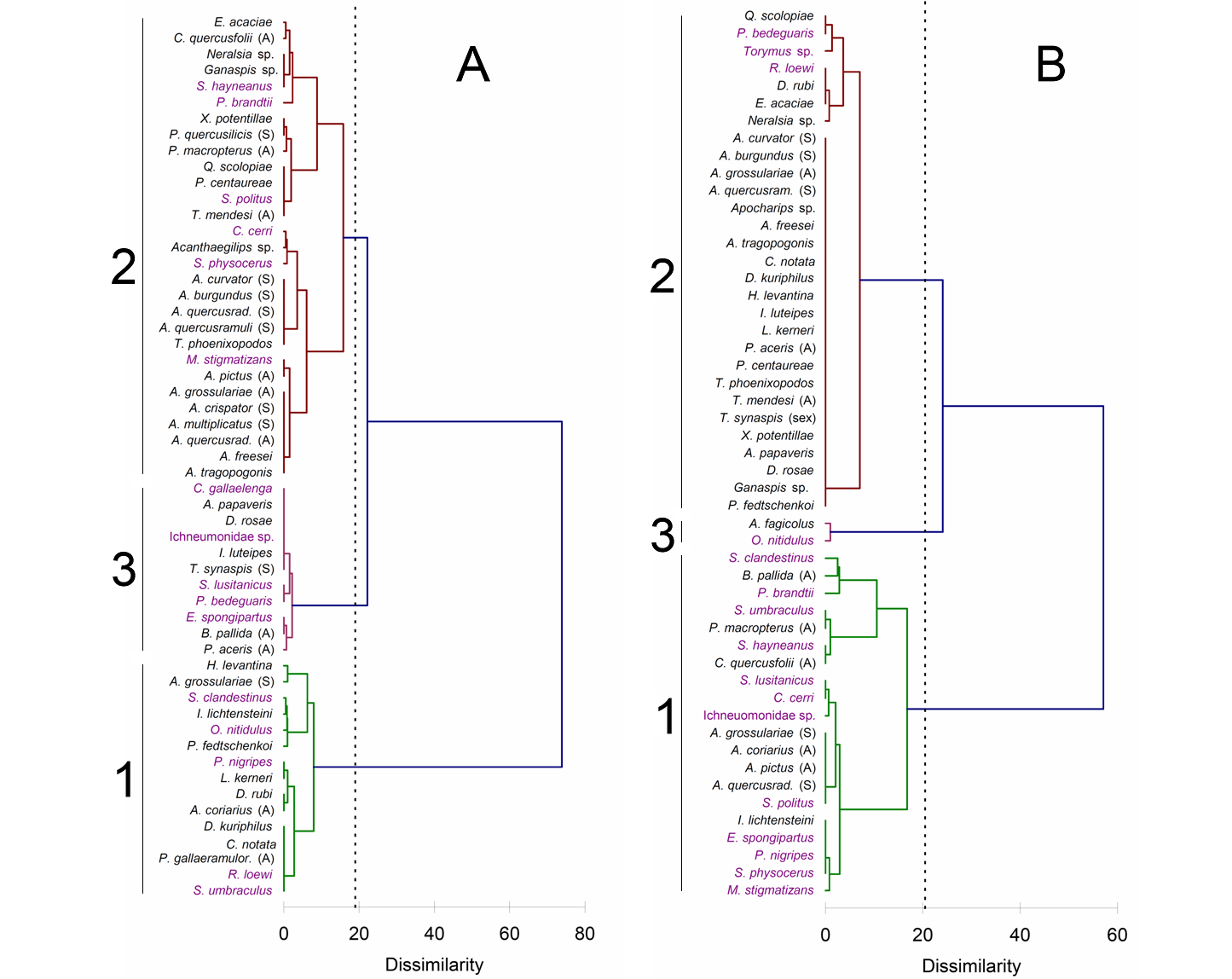

Supplement: Figure S3 — Dendrograms depicted by the cluster analyses (Ward method) based on the concentration ranks of the different metals recorded for each species. a, mandibles; b, ovipositor. The main groups recognized by the analysis are shown (the dissimilarity value which likely determines how many clusters best suit the data corresponds to the dashed line). Species whose names are in violet identify gall-invaders. For heterogonic species, (A) indicates the asexual form and (S) the sexual form. (TIF) [file pone.0070529.s003.tif]
