# Supplementary material for: Breaking up the Wall: Metal-Enrichment in Ovipositors, but Not in Mandibles, Co-Varies with Substrate Hardness in Gall-Wasps and Their Associates
Source: PLoS One. 2013 Jul 24;8(7):e70529. doi: 10.1371/journal.pone.0070529 (PMC3722128; doi:10.1371/journal.pone.0070529)
Supplement: Table S1 — Details of gall/substrate for each of the studied species, its rank, and references for hardness ranking. Species are listed in alphabetic order. Gall description in the “Emerging site” column refers to the mature gall. “–” identifies that for that species the organ used to emerge (mandibles) or to oviposit (ovipositor) was not analysed. (DOC) [file pone.0070529.s004.doc]

Table S1

| **Species** | **Emerging site** | **Oviposition site** | **Emerging substrate hardness** | **Ovipositing substrate hardness** | **References** |
| --- | --- | --- | --- | --- | --- |
| *Acanthaegilips* sp. | Host larval cuticle | - | 1 | - | [1] |
| *Aditrochus fagicolus* | - | Tree buds | - | 3 | [2] |
| *Andricus burgundus* (sexual) | Soft-juicy gall | Tree buds | 2 | 3 | [3] |
| *Andricus coriarius* (asexual) | Very hard gall with woody external layer | Tree buds | 4 | 3 | [3] |
| *Andricus crispator* (sexual) | Soft-juicy gall | - | 2 | - | [4] |
| *Andricus curvator* (sexual) | Soft-juicy gall | Tree buds | 2 | 3 | [3] |
| *Andricus grossulariae* (asexual) | Very hard gall with woody external layer | Tree flowers | 4 | 3 | [3] |
| *Andricus grossulariae* (sexual) | Soft-juicy gall | Tree buds | 2 | 3 | [3] |
| *Andricus multiplicatus* (sexual) | Soft-juicy gall | - | 2 | - | [4] |
| *Andricus pictus* (asexual) | Very hard gall with woody external layer | Tree stems | 4 | 3 | [3] |
| *Andricus quercusradicis* (asexual) | Very hard gall with woody external layer | Tree roots | 4 | - | [3] |
| *Andricus quercusradicis* (sexual) | Dry-hard gall without woody external layer | Tree stems | 3 | 3 | [3] |
| *Andricus quercusramuli* (sexual) | Soft-juicy gall | Tree buds | 2 | 3 | [3] |
| *Apocharips* sp. | - | Host larval cuticle | - | 1 | [5] |
| *Aulacidea freesei* | Dry-hard gall without woody external layer | Herb stems | 3 | 2 | [3] |
| *Aulacidea tragopogonis* | Dry-hard gall without woody external layer | Herb stems | 3 | 2 | [3] |
| *Aylax papaveris* | Dry-hard gall without woody external layer | Herb fruits | 3 | 2 | [3] |
| *Biorhiza pallida* (asexual) | Very hard gall with woody external layer | Tree roots | 4 | 3 | [3] |
| *Callaspidia notata* | Host larval cuticle | Host larval cuticle | 1 | 1 | [6] |
| *Cecinothofagus gallaelenga* | Dry-hard gall without woody external layer | - | 3 | - | [7] |
| *Ceroptres cerri* | Dry-hard gall without woody external layer | Immature host gall | 3 | 3 | [3] |
| *Cynips quercusfolii* (asexual) | Dry-hard gall without woody external layer | Tree leaves | 3 | 3 | [3] |
| *Diastrophus rubi* | Dry-hard gall without woody external layer | Arbust stems | 3 | 3 | [3] |
| *Diplolepis rosae* | Very hard gall with woody external layer | Arbust fruits, buds and leaves | 4 | 3 | [3] |
| *Dryocosmus kuriphilus* | Soft-juicy gall | Tree buds and leaves | 2 | 3 | [8] |
| *Eschatocerus acaciae* | Dry-hard gall without woody external layer | Tree twigs | 3 | 3 | [9] |
| *Eupelmus spongipartus* | Very hard gall with woody external layer | Immature host gall | 4 | 4 | [3] |
| *Ganaspis* sp. | Host larval cuticle | Host larval cuticle | 1 | 1 | [6] |
| *Hedickiana levantina* | Dry-hard gall without woody external layer | Herb stems | 3 | 2 | [3] |
| Ichneumonidae sp. | Very hard gall with woody external layer | Immature host gall | 4 | 4 | [3] |
| *Iraella luteipes* | Dry-hard gall without woody external layer | Herb stems | 3 | 2 | [3] |
| *Isocolus lichtensteini* | Dry-hard gall without woody external layer | Herb stems | 3 | 2 | [3] |
| *Liposthenes kerneri* | Dry-hard gall without woody external layer | Herb fruits | 3 | 2 | [3] |
| *Megastigmus stigmatizans* | Very hard gall with woody external layer | Mature host gall | 4 | 4 | [10] |
| *Neralsia* sp. | Host larval cuticle | Host larval cuticle | 1 | 1 | [6] |
| *Ormyrus nitidulus* | Very hard gall with woody external layer | Mature host gall | 4 | 4 | [3] |
| *Panteliella fedtschenkoi* | Dry-hard gall without woody external layer | Herb stems | 3 | 2 | [3] |
| *Parnips nigripes* | Dry-hard gall without woody external layer | Immature host gall | 3 | 2 | [11] |
| *Pediaspis aceris* (asexual) | Very hard gall with woody external layer | Tree roots | 4 | 3 | [3] |
| *Periclistus brandtii* | Very hard gall with woody external layer | Mature host gall | 4 | 3 | [3] |
| *Phanacis centaureae* | Dry-hard gall without woody external layer | Herb stems | 3 | 2 | [3] |
| *Plagiotrochus gallaeramulorum* (asexual) | Very hard gall with woody external layer | - | 4 | - | [3] |
| *Plagiotrochus quercusilicis* (sexual) | Soft-juicy gall | - | 2 | - | [3] |
| *Pseudoneuroterus macropterus* (asexual) | Very hard gall with woody external layer | Tree stems | 4 | 3 | [3] |
| *Pteromalus bedeguaris* | Dry-hard gall without woody external layer | Immature host gall | 4 | 3 | [12] |
| *Qwaqwaia scolopiae* | Dry-hard gall without woody external layer | Tree buds | 3 | 3 | [13] |
| *Rhoophilus loewi* | Dry-hard gall without woody external layer | Immature host gall | 3 | 3 | [14] |
| *Saphonecrus lusitanicus* | Very hard gall with woody external layer | Mature host gall | 4 | 3 | [3] |
| *Synergus clandestinus* | Very hard gall with woody external layer | Mature host gall | 4 | 3 | [3] |
| *Synergus hayneanus* | Very hard gall with woody external layer | Immature host gall | 4 | 3 | [3] |
| *Synergus physocerus* | Dry-hard gall without woody external layer | Immature host gall | 3 | 3 | [3] |
| *Synergus umbraculus* | Very hard gall with woody external layer | Immature host gall | 4 | 3 | [3] |
| *Synophrus politus* | Very hard gall with woody external layer | Immature host gall | 4 | 3 | [3] |
| *Timaspis phoenixopodos* | Dry-hard gall without woody external layer | Herb stems | 3 | 2 | [3] |
| *Torymus* sp. | - | Mature host gall | - | 4 | Unpublished data |
| *Trigonaspis mendesi* (asexual) | Dry-hard gall without woody external layer | Tree buds | 3 | 3 | [3] |
| *Trigonaspis synaspis* (sexual) | Soft-juicy gall | Tree stems | 2 | 3 | [3] |
| *Xestophanes potentillae* | Dry-hard gall without woody external layer | Herb stems | 3 | 3 | [3] |

**References Table S1**

1. Ronquist F, Hanson P, Buffington M, Fontal-Cazalla F, Ros-Farrè P (2006) Familia Figitidae. In: Hanson P, Gauld A (editors). Hymenoptera de la Región Neotropical. Gainesville: Mem Am Entomol Inst 77. pp. 280-293.
2. Nilsson M, Corley JC, Anderbrant O (2011) Factors affecting success of galls of *Aditrochus coihuensis* (Hymenoptera: Pteromalidae). Revista de la Sociedad Entomológica Argentina 70: 337-346.
3. Nieves-Aldrey JL (2001) Hymenoptera, Cynipidae. In: Fauna Ibérica, vol. XVI. Ramos, MA et al., editors. Madrid: Museo Nacional de Ciencias Naturales (CSIC). 636 pp.
4. Melika G (2006) Gall Wasps of Ukraine. Cynipidae. Vestnik zoologii, supplement 21, vol. 1–2. pp.1–644.
5. Paretas-Martínez J, Arnedo MA, Melika G, Selfa J, Seco-Fernández MV, et al. (2007) Phylogeny of the parasitic wasp subfamily Charipinae (Hymenoptera, Cynipoidea, Figitidae). Zool Scr 36: 153-172.
6. Ronquist F (1999) Phylogeny, classification and evolution of the Cynipoidea. Zool Scr 28:139-164.
7. Nieves-Aldrey JL, Liljeblad J, Hernandez Nieves M, Grez A, Nylander JAA (2009) Revision and phylogenetics of the genus Paraulax Kieffer (Hymenoptera, Cynipidae) with biological notes and description of a new tribe, a new genus, and five new species. Zootaxa 2200: 1-40.
8. Cooper WR, Rieske LK, (2010) Gall structure affects ecological associations of *Dryocosmus kuriphilus* (Hymenoptera: Cynipidae). Environ Entomol 39: 787-797.
9. Ronquist F, Liljeblad J (2001) Evolution of the gall wasp–host plant association. Evolution 55: 2503-2522.
10. Nieves-Aldrey JL, Hernández Nieves M, Gómez JF (2008) Larval morphology and biology of three European species of *Megastigmus* (Hymenoptera, Torymidae, Megastigminae) parasitoids of gall wasps, including a comparison with the larvae of two seed-infesting species. Zootaxa 1746: 44-60.
11. Ronquist F, Nieves-Aldrey JL (2001) A new subfamily of Figitidae (Hymenoptera, Cynipoidea). Zool J Linn Soc 133: 483-494.
12. Gómez JF, Nieves-Aldrey JL (2012) Notes on the larval morphology of Pteromalidae (Hymenoptera: Chalcidoidea) species parasitoids of gall wasps (Hymenoptera: Cynipidae) in Europe. Zootaxa 3189: 39-55.
13. Liljeblad J, Nieves-Aldrey JL, Nesser S, Melika G (2011) Adding another piece to the puzzle: the description of a South African gall wasp and a new tribe (Hymenoptera: Cynipidae). Zootaxa 2806: 35-52.
14. Van Noort S, Stone G, Whitehead VB, Nieves-Aldrey JL (2007) Biology of Rhoophilus loewi (Hymenoptera: Cynipoidea: Cynipidae), with implications for the evolution of inquilinism in gall wasps Biol J Linn Soc Lond 90: 153-17.
