# Supplementary material for: Breaking up the Wall: Metal-Enrichment in Ovipositors, but Not in Mandibles, Co-Varies with Substrate Hardness in Gall-Wasps and Their Associates
Source: PLoS One. 2013 Jul 24;8(7):e70529. doi: 10.1371/journal.pone.0070529 (PMC3722128; doi:10.1371/journal.pone.0070529)
Supplement: Table S2 — Trends from outer to inner part of the organs as obtained after line-scan. Species are listed in alphabetic order. Only significant models at P<0.05 are reported (in bold if R2≥0.5). “–”: organ not analyzed. Empty cell: no metals found or non-significant regressions. (DOC) [file pone.0070529.s005.doc]

Table S2

| **Species** | **Mandibles** | **Ovipositor** |
| --- | --- | --- |
| *Acanthaegilips* sp. | **Zn: sigmoid**, Cu: linear | - |
| *Aditrochus fagicolus* | - |  |
| *Andricus burgundus* (sexual) | **Zn: sigmoid** |  |
| *Andricus coriarius* (asexual) | **Zn: sigmoid**, Cu: linear | Mn: linear |
| *Andricus crispator* (sexual) | **Zn: sigmoid** | - |
| *Andricus curvator* (sexual) | **Zn: sigmoid** |  |
| *Andricus grossulariae* (asexual) | **Zn: sigmoid** |  |
| *Andricus grossulariae* (sexual) | **Zn: sigmoid**, Cu: linear | Mn: linear |
| *Andricus multiplicatus* (sexual) | **Zn: sigmoid** | - |
| *Andricus pictus* (asexual) | **Zn: sigmoid** | Mn: linear |
| *Andricus quercusradicis* (asexual) | **Zn: sigmoid** | - |
| *Andricus quercusradicis* (sexual) | **Zn: sigmoid** | Mn: linear |
| *Andricus quercusramuli* (sexual) | Zn: **sigmoid** |  |
| *Apocharips* sp. | - |  |
| *Aulacidea freesei* | **Zn: sigmoid** |  |
| *Aulacidea tragopogonis* | **Zn: sigmoid** |  |
| *Aylax papaveris* | **Zn: sigmoid** |  |
| *Biorhiza pallida* (asexual) | **Zn: sigmoid**, Cu: linear | Mn: linear |
| *Callaspidia notata* | **Zn: sigmoid**, Mn: linear, Cu: linear |  |
| *Cecinothofagus gallaelenga* | **Zn: sigmoid** | - |
| *Ceroptres cerri* | **Zn: sigmoid**, Cu: linear |  |
| *Cynips quercusfolii* (asexual) | **Zn: sigmoid**, Mn: linear | Mn: linear |
| *Diastrophus rubi* | **Zn: sigmoid**, Cu: linear |  |
| *Diplolepis rosae* | **Zn: sigmoid** |  |
| *Dryocosmus kuriphilus* | **Zn: sigmoid**, Cu: linear |  |
| *Eschatocerus acaciae* | **Zn: sigmoid**, **Mn: linear**, Cu: linear |  |
| *Eupelmus spongipartus* | **Zn: sigmoid, Cu: linear** |  |
| *Ganaspis* sp. | **Zn: sigmoid**, **Mn: linear,** Cu: linear |  |
| *Hedickiana levantina* | **Zn: sigmoid, Mn: linear**, Cu: linear |  |
| Ichneumonidae sp. | **Zn: sigmoid** | Mn: linear |
| *Iraella luteipes* | **Zn: sigmoid** |  |
| *Isocolus lichtensteini* | **Zn: sigmoid, Mn: linear,** **Cu: linear** | Mn: linear |
| *Liposthenes kerneri* | **Zn: sigmoid, Mn: linear,** Cu: linear |  |
| *Megastigmus stigmatizans* | **Zn: sigmoid** | **Zn: sigmoid,** Mn: linear |
| *Neralsia* sp. | **Zn: sigmoid, Mn: linear**, Cu: linear |  |
| *Ormyrus nitidulus* | **Zn: sigmoid**, Mn: linear, Cu: linear | **Zn: sigmoid,** Cu: linear |
| *Panteliella fedtschenkoi* | **Zn: sigmoid**, Mn: linear, Cu: linear |  |
| *Parnips nigripes* | **Zn: sigmoid**, Cu: linear | Mn: linear |
| *Pediaspis aceris* (asexual) | **Zn: sigmoid**, Cu: linear |  |
| *Periclistus brandtii* | **Zn: sigmoid**, Mn: linear, Cu: linear |  |
| *Phanacis centaureae* | **Zn: sigmoid** |  |
| *Plagiotrochus gallaeramulorum* (asexual) | **Zn: sigmoid** Mn: linear, Cu: linear | - |
| *Plagiotrochus quercusilicis* (sexual) | **Zn: sigmoid**, Mn: linear | - |
| *Pseudoneuroterus macropterus* (asexual) | **Zn: sigmoid**, Mn: linear |  |
| *Pteromalus bedeguaris* | **Zn: sigmoid**, Mn: linear | **Zn: sigmoid** |
| *Qwaqwaia scolopiae* | **Zn: sigmoid**, Mn: linear | Zn: linear, Cu: linear |
| *Rhoophilus loewi* | **Zn: sigmoid**, Mn: linear, Cu: linear | Cu: linear |
| *Saphonecrus lusitanicus* | **Zn: sigmoid** | Mn: linear, Cu: linear |
| *Synergus clandestinus* | **Zn: sigmoid,** Mn: linear, Cu: linear | Zn: linear, **Mn: linear**, Cu: linear |
| *Synergus hayneanus* | **Zn: sigmoid,** Mn: linear, Cu: linear |  |
| *Synergus physocerus* | **Zn: sigmoid**, Cu: linear |  |
| *Synergus umbraculus* | **Zn: sigmoid,** Mn: linear, Cu: linear | Mn: linear |
| *Synophrus politus* | **Zn: sigmoid** | Mn: linear |
| *Timaspis phoenixopodos* | **Zn: sigmoid** |  |
| *Torymus* sp. | - | **Zn: sigmoid** |
| *Trigonaspis mendesi* (asexual) | **Zn: sigmoid** |  |
| *Trigonaspis synaspis* (sexual) | **Zn: sigmoid** |  |
| *Xestophanes potentillae* | **Zn: sigmoid**, Mn: linear |  |
