# Supplementary material for: Breaking up the Wall: Metal-Enrichment in Ovipositors, but Not in Mandibles, Co-Varies with Substrate Hardness in Gall-Wasps and Their Associates
Source: PLoS One. 2013 Jul 24;8(7):e70529. doi: 10.1371/journal.pone.0070529 (PMC3722128; doi:10.1371/journal.pone.0070529)
Supplement: Table S3 — Mann-Whitney contrasts performed to roughly correct the results of the logistic ordinal regressions for phylogenetic relationships among species. In brackets, close to each metal tested, there are the compared medians. (DOC) [file pone.0070529.s006.doc]

Table S3

| **Contrast** | **Metal** | **Mann-Whitney test** |
| --- | --- | --- |
| Gall-invaders in substrate 3 × gall-inducers in substrate 3 (all groups) | Zn (0 vs. 0) | U=100.5, n_1_=11, n=19, P=0.81 |
|  | Mn (0 vs. 1) | U=68.5, n_1_=11, n=19, P=0.14 |
|  | Cu (0 vs. 0) | U=89, n_1_=11, n=19, P=0.67 |
| Gall-inducers in substrate 2 × gall-inducers in substrate 3 (Cynipidae) | Zn (0 vs. 0) | U=110, n_1_=11, n=19, P<0.0001 |
|  | Mn (0 vs. 0) | U=137, n_1_=11, n=19, P=0.05 |
|  | Cu (0 vs. 0) | U=126.5, n_1_=11, n=19, P=0.28 |
| Gall-invaders × gall-inducers (Cynipidae) | Zn (0 vs. 0) | U=124, n_1_=9, n=30, P<0.0001 |
|  | Mn (2 vs. 0) | U=64, n_1_=9, n=30, P<0.0001 |
|  | Cu (1 vs. 0) | U=76, n_1_=9, n=30, P<0.0001 |
| Gall-invaders (within Cynipidae) × gall-invaders (outside Cynipidae) | Zn (0 vs. 1) | U=17.5, n_1_=7, n=9, P=0.06 |
|  | Mn (2 vs. 1) | U=52, n_1_=7, n=9, P=0.03 |
|  | Cu (1 vs. 0) | U=38.5, n_1_=7, n=9, P=0.55 |
| Non-gallers × gall-inducers (all groups) | Zn (0 vs. 0) | U=58, n_1_=4, n=31, P=0.43 |
|  | Mn (0 vs. 0) | U=46, n_1_=4, n=31, P=0.67 |
|  | Cu (0 vs. 0) | U=69, n_1_=4, n=31, P=0.90 |
